# Pediatric Forearm Refracture with Intramedullary Nail Bending In Situ: Options for Treatment

**DOI:** 10.7759/cureus.6744

**Published:** 2020-01-22

**Authors:** Vadym Zhamilov, Ali Reisoglu, Can Doruk Basa, Ismail Eralp Kacmaz, Haluk Agus

**Affiliations:** 1 Orthopaedics, Tepecik Training and Research Hospital, Izmir, TUR

**Keywords:** pediatric forearm fracture, intarmedullary nail, refracture

## Abstract

In this study, we present the case of a nine-year-old male patient who had initially presented to the emergency department with a right both-bone forearm fracture. He was treated with closed reduction and long-arm casting. The cast was applied for six weeks and then replaced with a short-arm cast for two weeks. The patient returned with a both-bone forearm refracture one and a half months after the removal of the cast. Surgical treatment was initiated and an intramedullary nail fixation was applied. The patient sustained a new trauma five months postoperatively. The condition was diagnosed to be a refracture of the both-bone forearm with an intramedullary nail in situ. Closed reduction was performed, but an acceptable level of reduction was not achieved. Subsequently, intramedullary nails were replaced with new nails. At the one year follow-up, the patient was observed to have a full range of motion and reported no pain or muscle weakness.

## Introduction

The incidence of pediatric forearm shaft fractures is on an upward trend [1]. It is estimated that the forearm shaft fractures account for 6-10% of children’s fractures [2]. Although most of these fractures can be treated by closed reduction and casting, up to 25% of forearm fractures can be displaced during the follow-up and may need a second intervention [3]. If closed reduction fails, surgical intervention may be required. Internal fixation with elastic intramedullary nailing has become the treatment option of choice for forearm fractures in children [4,5]. The incidence of refractures of forearm fractures with an intramedullary nail in situ is very rare, and there are no formal guidelines for the management of these fractures [6].

## Case presentation

**Figure 1:**
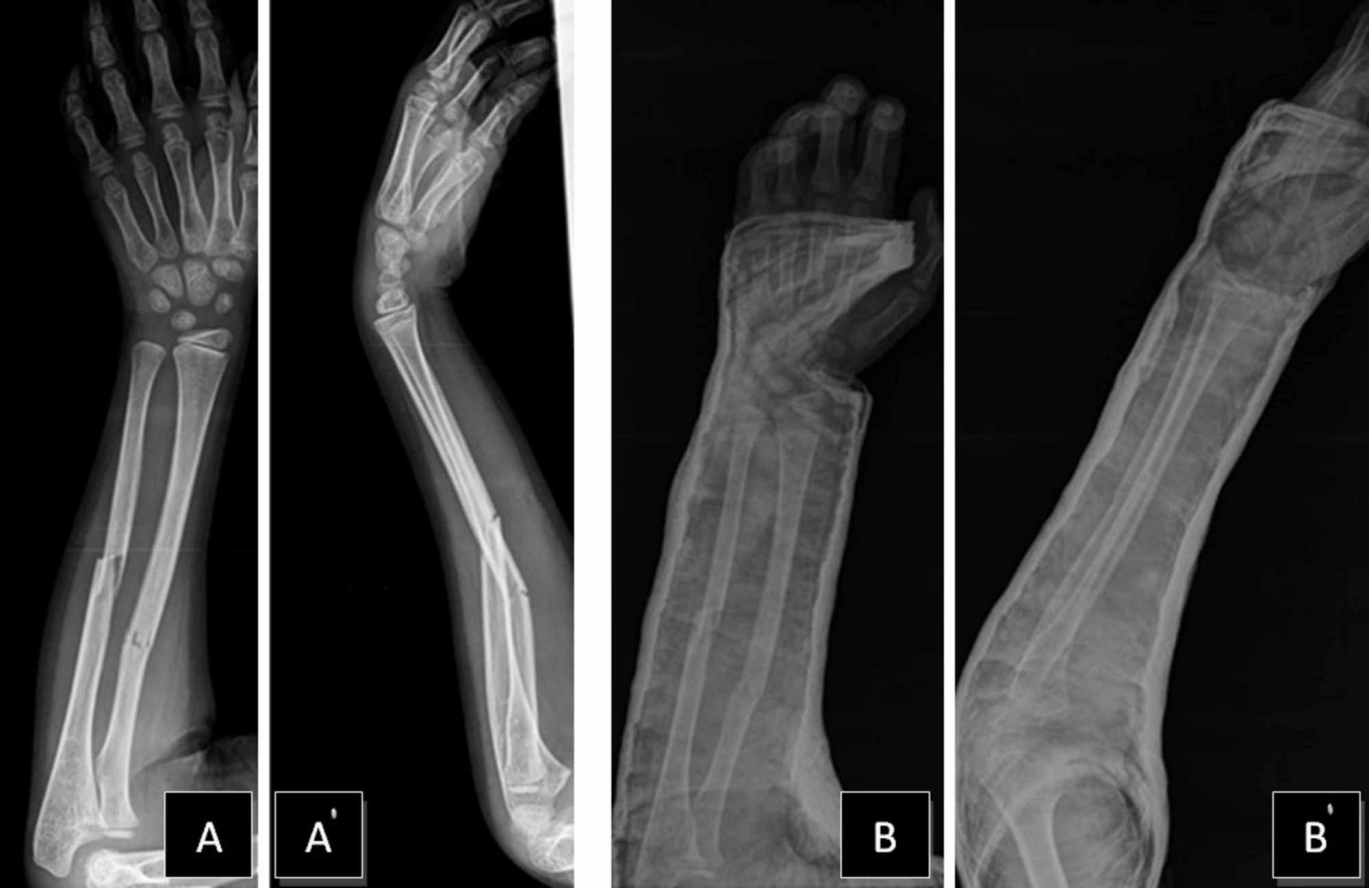
Imaging results of the patient's right forearm fracture at initial visit to the clinic A, A' – AP and lateral views of right forearm fracture B, B' – AP and lateral views of acceptable alignment after closed reduction and casting

**Figure 2:**
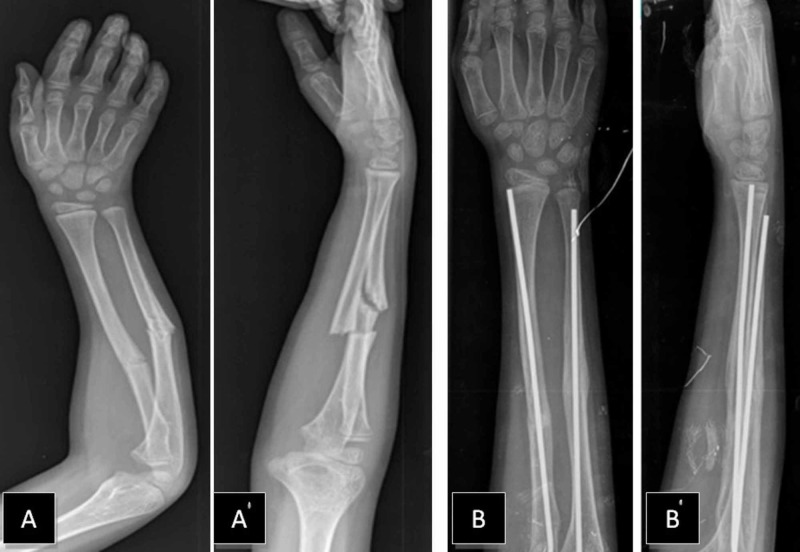
First refracture and postoperative views after open reduction and intramedullary nail fixation A, A' – AP and lateral views of right forearm refracture B, B' – AP and lateral postoperative views after open reduction and intramedullary nail fixation (fifth-month post-op)

**Figure 3:**
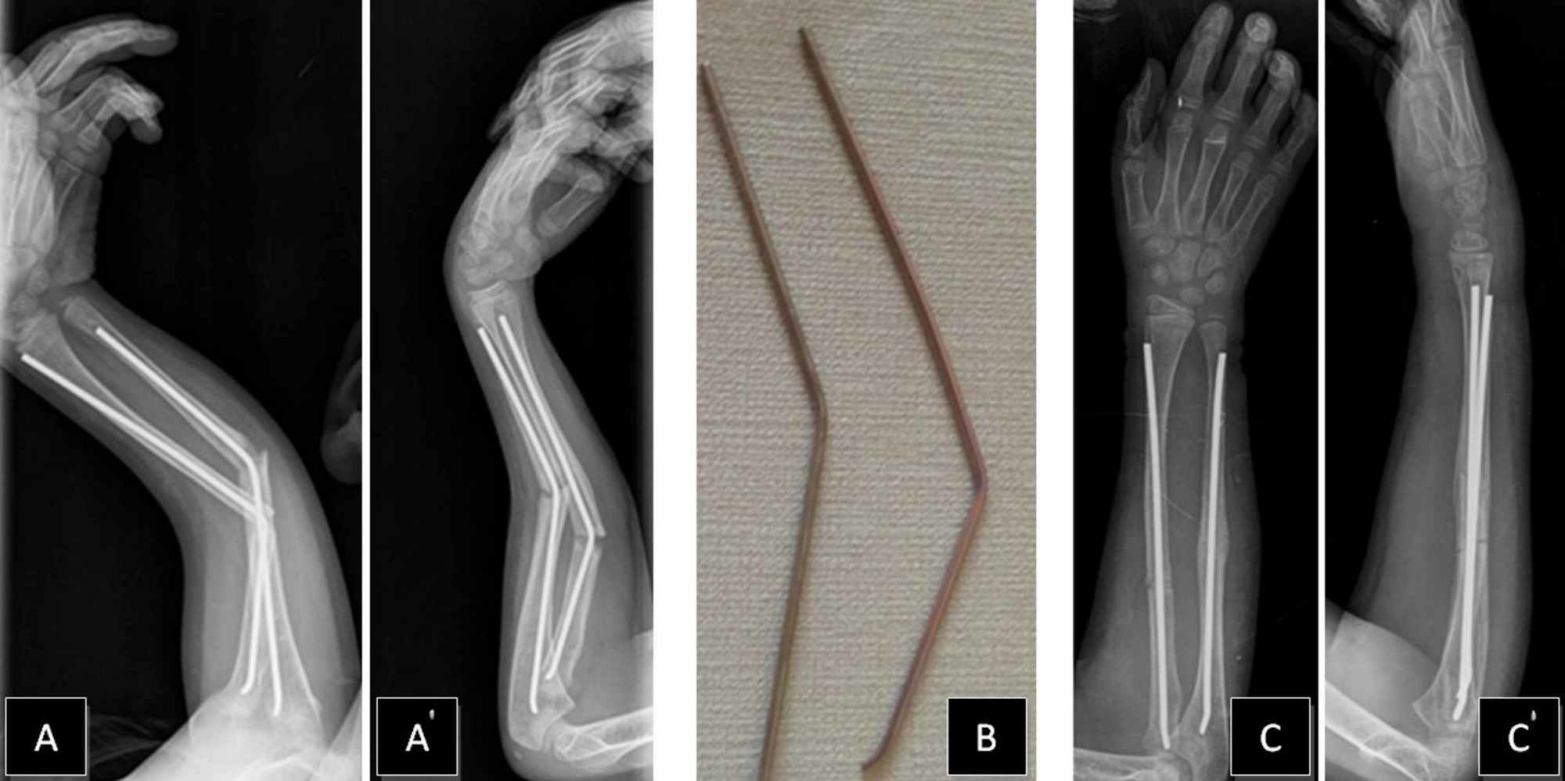
Forearm refracture with nails in situ and postoperative views A, A' – AP and lateral views of right forearm refracture with nails in situ (fifth-month post-op) B – bent nails after removal C, C' – AP and lateral postoperative views after closed reduction and intramedullary nail fixation

**Figure 4:**
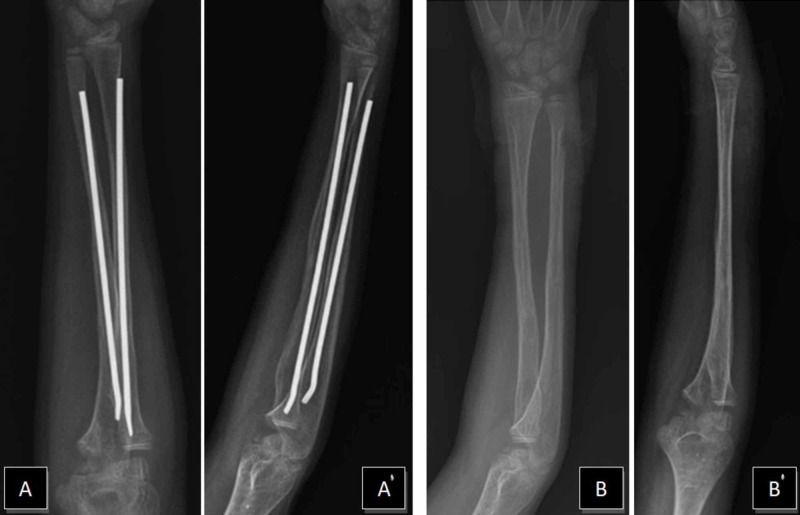
Views of forearm that shows complete healing and with removed nails A, A' – AP and lateral postoperative views (first-year post-op) B, B' – AP and lateral views of forearm with removed nails

A nine-year-old boy had initially presented to the clinic with a right both-bone middle shaft forearm fracture and treated with closed reduction and long-arm casting (Figure 1). A weekly clinical and radiographic review had been performed and, after eight weeks, radiological fracture healing had been established and the cast removed. Six weeks after the removal of the cast, the patient returned to our clinic with a refracture. A closed reduction was attempted under general anesthesia. However, acceptable alignment could not be achieved, and we proceeded directly to open reduction and internal fixation with Titanium Elastic Nail (TEN) (Figure 2). A postoperative cast was applied for two weeks. The patient sustained a new trauma in the fifth month postoperatively. A refracture of the both-bone forearm with an intramedullary nail in situ was diagnosed. In our clinic, the removal of the nail is generally performed one year after the surgery. The patient was investigated and no bone disease was found. Closed reduction was again performed, but an acceptable alignment could not be reached. Hence, the intramedullary nails were replaced with larger-sized nails (Figure 3). The older nails were in a bent condition. To remove the older nails, the forearm was opened using the incisions that had been made in the previous operation; the tips of the nails were located on the distal forearm and were subsequently removed. We had to apply only minimal force as it was possible to break the nail with re-manipulation. We made incisions on the distal side of the forearm at both-bone radius and ulna. The incision on the radius was performed in a retrograde fashion via entry point proximal to the distal physis, in the lateral plane and at the ulna on the medial side just proximal to the distal physeal line. The tips of the nails were grasped by nippers and straight-pulling was applied. Because of nail elasticity, there were no issues with the pull-out. New nails were inserted after removing both of the bent nails. The nails were inserted by tapping with a hammer. The size of removed nails was 3 mm and that of the new ones was 3.5 mm. The nails were cut just close to the bones and a long-arm cast was applied. At a follow-up after 12 months, clinical and radiological healing of the fracture was observed. The nails were removed in one year (Figure 4). At the first-year follow-up, the patient was observed to have a full range of motion.

## Discussion

The majority of pediatric forearm fractures are treated with closed reduction and casting [7]. Unacceptable reduction, segmental fractures, unstable fracture pattern, open fracture, loss of reduction, and compartment syndrome are considered as indications for surgical intervention in cases of diaphyseal fractures of the radius and ulna [8]. Recently, flexible intramedullary nailing has been widely performed for pediatric forearm fractures. Intramedullary fixation of the forearm fracture is a safe, effective, and accessible technique. Refracture following forearm fracture in children occurs in about 5% of the cases [9]. However, refracture with the intramedullary nail in situ is not common. In our case, the boy experienced a second refracture with the intramedullary nail in situ. Mittal et al. have reported a similar case, although their patient was 14 years old and the nail had been broken [10]. They suggested the removal and insertion of a new nail. We used the same technique in our patient. Fernandez et al. have reported the largest series of refractures with a nail in situ; however, five of their 14 patients fell within six weeks of the operation, and the fractures had not healed [11]. The rest of the patients had a refracture with the nail in situ. The authors have not provided detailed information about the treatment and consequences. Schmittenbecher's report discussed four patients with a refracture in the forearm with a nail in-situ, but they were treated with closed reduction. We attempted closed reduction for our patient, but it was unsuccessful. In cases of refractures of forearm fractures with intramedullary nails in situ that are reported in the literature, most of the patients were treated with closed reduction and nail replacement [11,13,14]. But some patients were treated with closed reduction leaving nails in situ, and applying plaster if a reduction was acceptable [6,12]. We had permission to try closed reduction first and, if acceptable reduction could not be reached, nail exchange could be used. The technique of removing bent nails from the forearm has not been described in the literature. Also, we were unable to find any information about the size of the nails. The diameter of the nails was 2/3 of the medullary canal, measured in the narrowest medullary cavity, and we also prebent the nails to restore radial bow. This information may be more important than currently considered, and we believe that it is important to highlight this factor.

More data should be made available about open or closed reduction as it can help make changes with the healing of fractures and provide clarity on nonunion after intramedullary nailing [15]. Schwarz at al. identified that refractures could occur because of delayed healing, which can be reduced by the prevention of healing disturbances [9]. We also need more knowledge about the levels of fracture in the forearm. Bould et al. reported that diaphyseal refracture rates are greater than metaphyseal [3]. It can be important because most of the refractures can statistically have the same level, and it can affect the treatment plan. Fernandez et al. have reported that the usage of an intramedullary nail is problematic for the distal diaphyseal portion of the forearm [11].

The special attention to proper surgical techniques for forearm refractures with the nail in situ would reduce the rates of complications. Egmond et al. performed a closed reduction of the refracture and an acceptable reduction was achieved [6]. But it was a refracture of the radius, and the ulna had no deformation. Mittal et al tried closed reduction; but after full correction, the ulnar nail was broken at the fracture site [10]. This complication is more difficult to manage because it is necessary to open the fracture line to remove the nail in such cases. Also, Muensterer et al. showed that the mechanical stability of the nails is significantly reduced if the nails bent to 21° [16]. Shahid et al. discussed a case where the distal site of radial nail insertion was opened and the intramedullary nail withdrawn until the bent part of the intramedullary nail was moved distally to the refracture line [17]. However, the nails have been getting shorter and the optimal three-point fixation gone. We believe that open or closed TEN-renailing with thicker nails, if possible, is a suit­able treatment for refractures with nails in situ.

## Conclusions

Intramedullary nail fixation is a minimally invasive technique for the primary and definitive management of forearm fractures. Refracture of both- bone forearm fractures with elastic stable intramedullary nails in situ is a rare complication of surgically treated pediatric forearm fractures. Closed reduction of a refracture is one of the treatment options. However, we conclude that TEN-renailing with open or closed technique is a better option for the treatment of pediatric forearm refracture with nails in situ.
